# Aortic arch debranching and extra-anatomic bypass for the surgical treatment of aortoesophageal fistula secondary to thoracic endovascular aortic repair

**DOI:** 10.3389/fbioe.2026.1664041

**Published:** 2026-02-13

**Authors:** Qun Lang, Lizhong Sun, Wei Liu, Kaitao Jian, Hao Peng, Yi Lin, Yu Xia

**Affiliations:** Department of Cardiovascular Surgery, Shanghai Delta Health Hospital, Shanghai, China

**Keywords:** antibiotic treatment, aortoesophageal fistula, clinical data, surgical treatment, thoracic endovascular aortic repair

## Abstract

**Background:**

Aortoesophageal fistula (AEF) is a relatively rare and life-threatening condition, and the optimal surgical treatment for secondary AEF following thoracic endovascular aortic repair (post-TEVAR AEF) remains controversial. This study aimed to summarize the clinical efficacy of aortic arch debranching combined with extra-anatomic bypass for the treatment of post-TEVAR AEF.

**Methods:**

The clinical data of 16 patients who underwent surgical treatment for post-TEVAR AEF at our institution from 30 June 2019 to 30 June 2024 were retrospectively reviewed. Aortic arch debranching and extra-anatomic aortic bypass under general anesthesia were performed for most patients. Empirical antibiotics were administered for 6–8 weeks. The acute and long-term outcomes were summarized.

**Results:**

Stent-related infection leading to AEF occurred at a median interval of 30 months after the initial TEVAR surgery. All patients presented with recurrent fever preoperatively; blood bacterial cultures were positive in nine patients (56.25%) and negative in seven patients (43.75%). The median operative time was 460.5 (433.5, 543.5) minutes, and the median intensive care unit stay was 7 (5.25, 31.75) days. No intraoperative mortality was observed in this cohort. During the follow-up period, three patients developed recurrent AEF accompanied by severe infection. Four patients died postoperatively, including one who died of thoracic aortic rupture and hemorrhage within 3 months postoperatively, and three others died of multiple organ failure at 4–10 weeks after surgery. The remaining 12 patients achieved favorable postoperative recovery without the need for prolonged antibiotic therapy.

**Conclusion:**

Aortic arch debranching and extra-anatomic bypass from the ascending aorta to the proximal abdominal aorta yields favorable acute and long-term outcomes for patients with post-TEVAR AEF.

## 1 Introduction

Thoracic endovascular aortic repair (TEVAR), a type of endoluminal aortic stent, has recently been developed for the standard treatment of patients with acute and chronic thoracic aortic pathologies (Czerny et al., 2021). Notably, with the widespread adoption of endoluminal aortic stent therapy, the occurrence of aortoesophageal fistula (AEF) following TEVAR (post-TEVAR AEF) has increased correspondingly, which remains a challenge for the use of TEVAR regarding post-TEVAR AEF (Dick et al., 2009; Uno et al., 2017). Post-TEVAR AEF is an extremely rare complication, with an incidence of only 1.5%–1.9% (Henrique et al., 2023; Rey Chaves et al., 2023). Currently, there are few large-scale studies reporting on the treatment strategies for this condition.

Traditionally, the presence of AEF is associated with a poor condition induced by acute exsanguination, persistent anemia, and recurrent sepsis (Eggebrecht et al., 2009; Schoel and Lagisetty, 2024). According to statistical data, the 1-year survival rate of patients treated with antibiotics or esophageal stents alone is merely 17%, whereas conservative management results in a 100% mortality rate within 1 year (Czerny et al., 2014). Thus, surgical intervention is recognized as the most effective therapeutic strategy for secondary AEF (Enomoto et al., 2020; Yoshitake et al., 2022; Lee et al., 2023). At present, post-TEVAR AEF is frequently complicated by thoracic infections, and reimplanting a stent in such an infected environment would be extremely detrimental to the control of future infections (Sugiyama et al., 2020). Consequently, many of these patients require lifelong antibiotic therapy after surgery (Canaud et al., 2014).

Thus, the infection risk induced by the surgical intervention urgently needs to be reduced. In this article, we present a retrospective review of 16 cases of post-TEVAR AEF, summarizing the clinical manifestations and surgical management strategies to optimize future clinical decision-making.

## 2 Methods

### 2.1 Patients

We retrospectively reviewed the clinical charts of 16 consecutive patients who were referred to Shanghai Delta Health Hospital for surgical intervention for post-TEVAR AEF between June 2019 and June 2024. All patients had undergone initial TEVAR at other institutions and subsequently developed AEF with stent-related infection. Due to the referral nature of the cohort, complete baseline data on the initial TEVAR were not fully available. Based on the available records, the initial indication for TEVAR was type B aortic dissection for the majority of the patients, while for a minority, it was a thoracic aortic aneurysm. All patients had proximal landing zones in zone Z2 or Z3. No patient had a history of prior aortic surgery before developing AEF. Among these patients, 15 were male and 1 was female. AEF developed after stent implantation, with a median time of 30 months (ranging from 2 weeks to 13 years). Additionally, clinical data, including the clinical symptoms, pre-existing comorbidities, preoperative antibiotic use, and classification of infectious bacteria, were all collected.

Clinical data were collected from patient records. All patients had provided written consent and agreed to have their data made available for research purposes. The local ethics committee approved this study (approval number SDH 2024 005).

### 2.2 Selection criteria

AEF was diagnosed based on a comprehensive assessment of three components: (1) a clear history of fever or other infectious manifestations, such as hematemesis or dysphagia; (2) radiological evidence, including the presence of a bubble shadow around the stent on aortic CT angiography or positive findings on PET-CT when AEF could not be diagnosed; (3) confirmation of esophageal fistula location and size via preoperative esophagoscopy. A positive blood culture was considered a supportive diagnostic criterion but was not mandatory, given the high rate of prolonged preoperative antibiotic use in this referral cohort.

### 2.3 Surgery procedure

**FIGURE 1:**
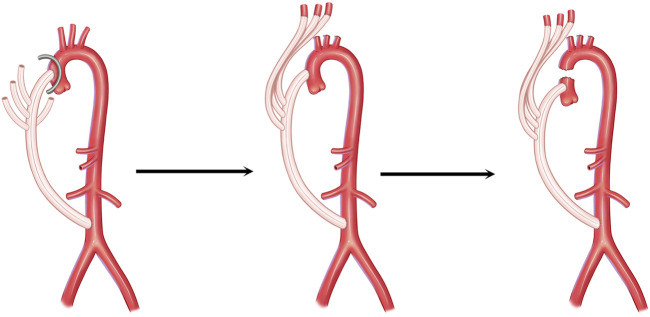
Schematic illustration of the two-stage surgical strategy for post-TEVAR infected AEF. The graft was anastomosed to the ascending aorta to establish the bypass from the ascending aorta to the abdominal aorta using a side-wall clamp.

A two-stage surgical strategy was utilized for all patients, as illustrated in Figure 1. The patient was placed in a supine position, and a midline thoraco-abdominal incision was created, extending from the suprasternal notch to the umbilicus. For patients requiring abdominal aortic reconstruction, the incision was further extended caudally to the pubis. The abdominal aorta was exposed via a transperitoneal approach. Systemic anticoagulation was achieved with intravenous administration of heparin (100 U/kg), targeting an activated clotting time (ACT) > 400 s to ensure adequate anticoagulation during vascular anastomosis.

#### 2.3.1 First-stage procedure: extra-anatomic bypass and aortic arch debranching

A side-biting (partially occluding) vascular clamp was applied to the infrarenal abdominal aorta, and a commercially available woven Dacron four-branched vascular graft (22 Fr–24 Fr, size individualized based on the preoperative CTA-measured aortic diameter) was anastomosed to the aortic wall in an end-to-side fashion using running 4-0 polypropylene sutures (Prolene, Ethicon). The graft was routed through the lesser omental sac, passed anterior to the esophagus and stomach, and delivered into the thoracic cavity via a transdiaphragmatic tunnel anterior to the heart and into the mediastinum.

**FIGURE 2:**
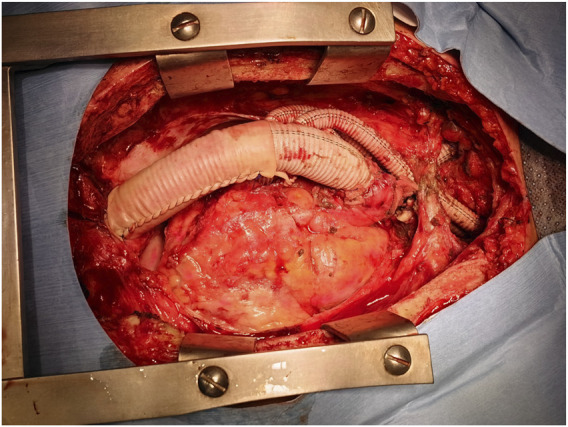
Intraoperative view of the completed extra-anatomic bypass graft during the first-stage procedure.

Subsequently, a median sternotomy was performed to expose the ascending aorta. A side-biting clamp was applied to the ascending aorta, and a multi-branch arch graft with three separate side arms was anastomosed to the ascending aortic wall in an end-to-side configuration using running 4-0 polypropylene sutures. The previously placed transdiaphragmatic abdominal graft was then coupled with the thoracic inflow graft in an end-to-end manner with the same suture material (Figure 2). Each aortic arch vessel (brachiocephalic artery, left common carotid artery, and left subclavian artery) was sequentially transected, mobilized, and individually reimplanted onto the corresponding side arms of the multi-branch graft to ensure balanced cerebral perfusion. Following the completion of revascularization, the distal ascending aorta was transected just proximal to the native aortic arch and graft takeoff. Hemostasis was meticulously secured, and the thoraco-abdominal incision and sternotomy were closed in a standard layered fashion with absorbable sutures for deep tissues and non-absorbable sutures for the skin.

#### 2.3.2 Second-stage procedure: infected stent excision and AEF repair

A left posterolateral thoracotomy was performed through the sixth or seventh intercostal space. The descending thoracic aorta was isolated and clamped above the diaphragmatic hiatus to minimize intraoperative bleeding. The native infected thoracic aorta and previously deployed endograft were opened and completely excised. Special care was taken to preserve the critical intercostal arterial feeders below the sixth thoracic vertebra (T6) as these vessels are vital for spinal cord perfusion, thereby minimizing the risk of spinal cord ischemia and postoperative paraplegia.

**FIGURE 3:**
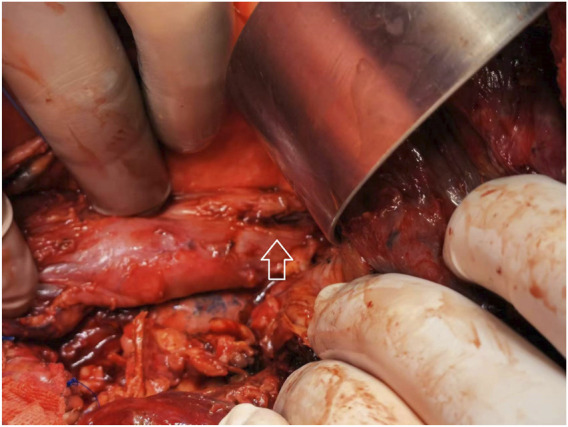
Intraoperative view of the infected aortoesophageal fistula. The white arrow indicates the site of the persistent esophageal fistula.

The AEF was carefully identified (Figure 3). For fistula repair, the esophageal defect was primarily closed with interrupted 4-0 polypropylene sutures, followed by buttressing with a vascularized intercostal muscle flap to augment tissue healing and reduce the recurrence risk. Multiple drainage and exclusion tubes were placed around the fistulous interface to facilitate postoperative fluid evacuation and infection control. In cases where the esophageal breach was not readily visualized, the thoracic cavity was filled with warm normal saline, and air was insufflated via an esophageal tube to localize the defect by observing air escape (bubbling) in the saline bath.

**FIGURE 4:**
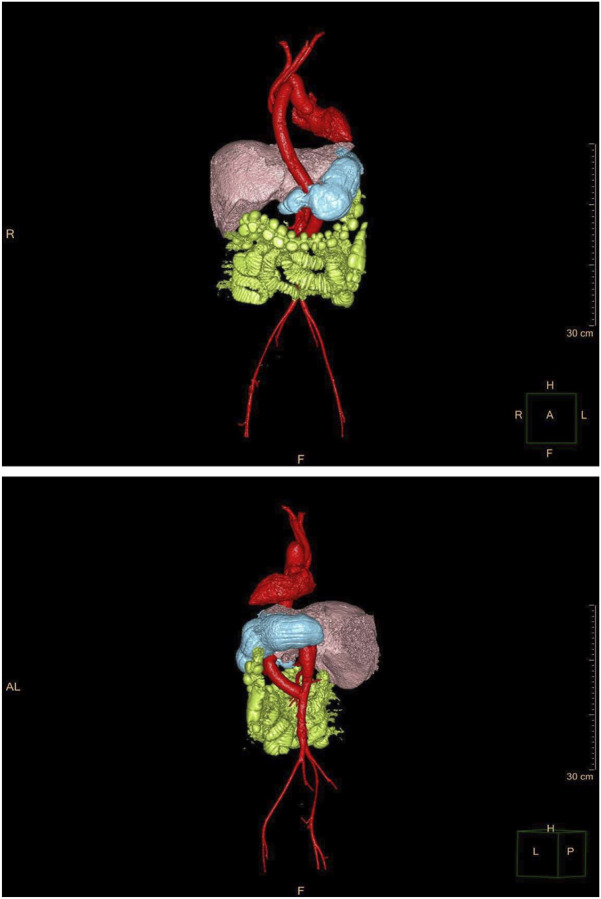
3D CT reconstruction images demonstrating the anatomical position of the extra-anatomic bypass graft (red) relative to adjacent organs (liver: pink; stomach: blue; intestine: green) after the first-stage procedure.

A schematic diagram of the postoperative graft routing is shown in Figure 4.

### 2.4 Hemodynamic analysis

To evaluate the hemodynamic rationality of the surgical strategy, we performed patient-specific hemodynamic simulations before and after the procedure. The fluid mechanics processing method for this procedure comprises preoperative and postoperative processing. In preoperative processing, the patient-specific anatomical data were acquired from preoperative aortic CTA scans, followed by image preprocessing to build and initialize a vascular fluid mechanics model. Postoperative CTA scans were obtained and processed using DetecModeling software (Boea Wisdom, Zhejiang, China) to update the model, simulate postoperative hemodynamics, and evaluate the effectiveness of the intervention.

### 2.5 Postoperative care and follow-up

Infection status was assessed using the white blood cell count, C-reactive protein level, erythrocyte sedimentation rate, and blood culture. Antibiotic use was individualized based on preoperative and intraoperative microbiological data. For patients with preoperative positive blood cultures and confirmed sensitivity results, postoperative antibiotics were continued using the same effective regimen that had controlled fever and infectious symptoms preoperatively. For patients with negative preoperative cultures but positive intraoperative cultures, postoperative antibiotics were adjusted according to the intraoperative culture sensitivity results. For patients with no available preoperative or intraoperative culture data, empirical broad-spectrum antibiotics were administered to cover Gram-positive bacteria, Gram-negative bacteria, and fungi. All patients received a standardized antibiotic course of 6–8 weeks postoperatively.

Intravenous nutrition was initiated within 1 week after surgery, and nutritional support was then gradually transitioned to intestinal tube feeding. After confirming the healing of the esophageal fistula through follow-up esophagography, feeding was resumed, and the chest drainage tube was gradually withdrawn every 2 weeks. The patient received total aortic CTA in the outpatient department at 6 months and 1 year after hospital discharge.

During follow-up, patients were considered to have recurrent AEF if they met all three of the following criteria: (1) persistent or recurrent massive purulent drainage in the thoracic cavity; (2) positive bacterial culture of the drainage fluid with the same pathogen as identified preoperatively; and (3) confirmation of an unhealed esophageal fistula.

Severe infection was defined as septic shock requiring vasopressor support to maintain vital signs despite adequate antibiotic therapy and fluid resuscitation.

### 2.6 Data analysis

Normality of continuous variables was assessed using the Shapiro–Wilk test. Data were presented as the mean ± standard deviation (SD) for normally distributed data or the median (Q1, Q3) for non-normally distributed data. Categorical data were presented as numbers (percentages). Kaplan–Meier survival analysis was performed using the “survminer” package in R version 4.3.3. The time origin was defined as the date of the surgical procedure. The primary endpoint for overall survival was all-cause death, with endpoint dates determined as the date of death for deceased patients; surviving patients were censored at the final follow-up date. Recurrence-free survival was defined as the time from surgery to the first occurrence of AEF recurrence or all-cause death, whichever came first.

## 3 Results

### 3.1 Baseline characteristics of patients

A total of 16 patients, including 15 male individuals and 1 female individual, were included in this study. The baseline characteristics of the patients are summarized in Table 1. The average age was 52.7 ± 6.7 years. Regarding the initial symptoms, fever was observed in all 16 patients (100%), and hematemesis occurred in five patients (31.25%). Three patients (18.75%) had dysphagia, a hissing sound, and black stools, respectively. Ten patients (62.5%) had a history of hypertension, three patients (18.75%) had type II diabetes, and one patient (6.25%) had an aortic root aneurysm. Bacterial culture was successful in nine patients, and seven of them were infected with Gram-negative bacteria (Table 2). AEF occurred with a median interval of 30 months (2.275, 81.75) after stent implantation. Notably, one patient underwent a Bentall operation due to the presence of an aortic root aneurysm, and this patient was the only person who required extracorporeal circulation during the surgery.

**TABLE 1:** Baseline preoperative characteristics of the included patients.

Variable	​	Value
Sex, n (%)		
​	Male	15 (93.75%)
	Female	1 (6.25%)
Age, years, mean ± SD	52.7 ± 6.7	
BMI, mean ± SD	23.4 ± 2.7	
Symptom, n (%)		
​	Fever	16 (100%)
	Dysphagia	1 (6.25%)
	Hematemesis	5 (31.25%)
	Dysphonia	1 (6.25%)
	Black stool	1 (6.25%)
Pre-existing comorbidity, n (%)		
​	Hypertension	10 (62.5%)
	Type II diabetes	3 (18.75%)
	Aortic root aneurysm	1 (6.25%)

SD, standard deviation.

**TABLE 2:** Preoperative infection of patients.

Variable	Value
Preoperative antibiotic use duration, months, median (Q1, Q3)	2 (1, 3.5)
Bacterial taxonomy, n (%)	
Gram-positive bacteria	3 (18.75%)
Gram-negative bacteria	7 (43.75%)
Fungus	1 (6.25%)
None	7 (43.75%)
Time range from stent implantation to infection, months, median (Q1, Q3)	30 (2.275, 81.75)

The total number of pathogen entries exceeds the number of patients because some patients had mixed infections with multiple organisms.

### 3.2 Acute and long-term outcomes

Table 3 shows that the surgery duration was 460.5 (433.5, 543.5) min, followed by a median intensive care unit (ICU) stay of 7 days (5.25, 31.75). After the surgery, the median hospital stay was 87.5 (61.5, 111) days, and the median fasting time was 75 (46, 98) days. The average duration of postoperative antibiotic administration was 7 weeks. All patients achieved effective infection control after receiving the individualized antibiotic regimen combined with surgical treatment. Notably, no patient required long-term suppressive antibiotic therapy during the follow-up period.

**TABLE 3:** Treatment status and initial interventional characteristics.

Variable	Value	
Surgery timing, min, median (Q1, Q3)	460.5 (433.5, 543.5)	
ICU stay, day, median (Q1, Q3)	7 (5.25, 31.75)	
AEF recurrence, n (%)	3 (18.75%)	
Postoperative complication, n (%)		
​	Cerebral ischemic stroke	1 (6.25%)
	Incision infection	4 (25%)
	Mediastinal infection	3 (18.75%)
	Artificial blood vessel rupture	2 (12.50%)
	Aortic sinus dissection	1 (6.25%)
	Paraplegia	1 (6.25%)
Clavien–Dindo grade		
​	1	6 (37.50%)
	2	1 (6.25%)
	3	3 (18.75%)
	4	2 (12.50%)
	5	4 (25%)
Follow-up time, day, median (Q1, Q3)	​	707 (453, 1127.5)
Hospital stay, day, median (Q1, Q3)	87.5 (61.5, 111)	
Fasting time, day, median (Q1, Q3)	75 (46, 98)	

ICU, intensive care unit; SD, standard deviation. The follow-up time was calculated for the remaining 12 patients. One patient had concurrent mediastinal infection, artificial blood vessel rupture, and paraplegia; thus, the total number of complication events exceeds the number of patients.

Postoperative complications were observed in 10 patients (62.5%, 10/16), including 1 case of cerebral ischemic stroke, 4 cases of incision infection, 1 case of artificial blood vessel rupture, 2 cases of mediastinal infection, 1 case of type A aortic dissection, and 1 case with concurrent artificial blood vessel rupture, mediastinal infection, and paraplegia. Four patients, namely, the one with the cerebral ischemic stroke, two with mediastinal infection, and the one with concurrent artificial blood vessel rupture, mediastinal infection, and paraplegia, ultimately died. Three of them succumbed to multiple organ failure, and one died from thoracic aortic rupture and hemorrhage. Notably, three out of the four fatal cases were associated with AEF recurrence (recurrence time: 2–6 weeks postoperatively). According to the Clavien–Dindo classification, the incidence of complications of grade 3 or higher was 56.3%, including four patients with grade 5, two patients with grade 4, and three patients with grade 3 complications (Table 3). The four patients who died were all of Clavien–Dindo grade 5.

### 3.3 Follow-up

**FIGURE 5:**
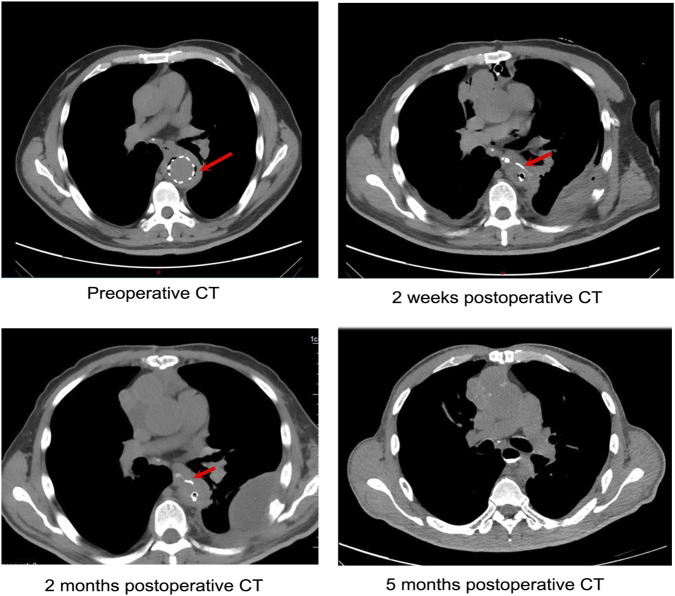
Esophagography of preoperative, 2 weeks, 2 months, and 5 months after operation. Arrows indicate the extravasation of the contrast agent.

**FIGURE 6:**
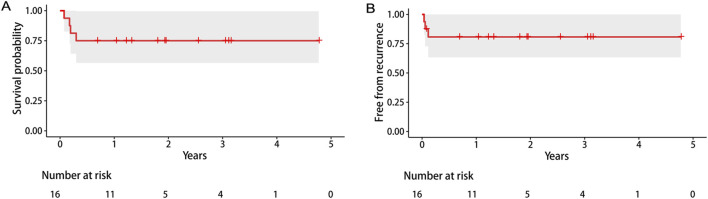
Kaplan–Meier survival curves of overall survival **(A)** and recurrence-free survival **(B)**.

Figure 5 shows the CT scans from preoperative to 5 months post-surgery of a typical patient. The preoperative CT scan revealed characteristic infection-related air bubbles. Esophagography indicated the extravasation of the contrast agent in the esophagus 2 weeks and 2 months after the operation. Esophagography performed 5 months after the operation showed no extravasation of contrast media, indicating that the esophageal fistula had healed. The median follow-up time for the remaining 12 patients was 707 (453, 1127.5) days (Table 3). The overall survival rate was 75% (95% confidence interval: 56.5%, 99.5%), and the recurrence-free survival rate was 80.8% (95% confidence interval: 63.4%, 100%) (Figure 6).

### 3.4 Hemodynamic outcomes

**FIGURE 7:**
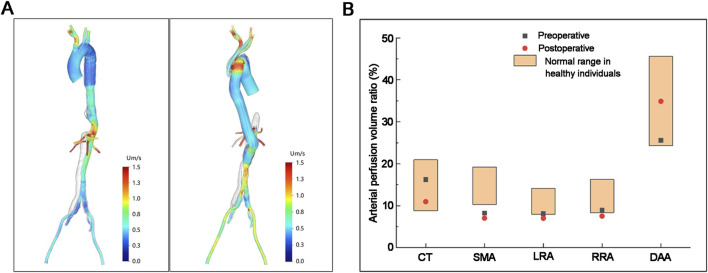
Postoperative hemodynamic characteristics of the extra-anatomic bypass graft. **(A)** Systolic peak blood flow streamline maps of the thoracic–abdominal aortic segment (left: preoperative; right: postoperative). The color bar indicates blood flow velocity (unit: m/s). **(B)** Perfusion volume ratio of key visceral and peripheral arteries (CT, celiac trunk; SMA, superior mesenteric artery; LRA, left renal artery; RRA, right renal artery; DDA, distal abdominal aorta–iliac artery). The box plot represents the normal perfusion range of healthy individuals; black squares indicate preoperative values, and red dots indicate postoperative values.

To verify the hemodynamic feasibility of the surgical strategy, patient-specific hemodynamic simulations were performed pre- and postoperatively (Figure 7). For blood flow stability (Figure 7A), the postoperative streamline map of the thoracic–abdominal artificial graft segment showed uniform blood flow distribution and no obvious swirling flow, with velocity values ranging from 0.0 to 1.5 m/s. This indicated that the extra-anatomic bypass design maintained stable laminar flow in the graft, avoiding hemodynamic disturbance. The perfusion volumes of the celiac trunk, left renal artery, and right renal artery slightly decreased postoperatively, but all values remained within the normal range for healthy individuals. The perfusion volume of the superior mesenteric artery was slightly reduced postoperatively. However, it was consistent with its preoperative level, and both values were below the healthy range. The perfusion volume of the distal abdominal aorta–iliac artery increased postoperatively, indicating improved blood supply to the lower extremities (Figure 7B). Overall, the hemodynamic analysis confirmed that the surgical approach maintained physiological organ perfusion while ensuring stable blood flow in the graft.

## 4 Discussion

Post-TEVAR AEF typically occurs from 1 week to several years after interventional stenting, and it is a relatively rare yet life-threatening condition (Takeno et al., 2020; Watanabe et al., 2020). Currently, there is no consensus regarding surgical intervention and surgical management (Buerger et al., 2020; Enomoto et al., 2020). In this study, aortic arch debranching and extra-anatomic aortic bypass under general anesthesia were performed to treat post-TEVAR AEF, achieving a favorable prognosis.

Currently, surgical treatment represents the most effective strategy for post-TEVAR AEF, although outcomes remain only marginally satisfactory in clinical practice (Okubo et al., 2021; Jin et al., 2024). Traditionally, the implantation of thoracic aortic stents often necessitates the proximal anchor to be located in zone Z3 or even in zones and Z2 (Mestres et al., 2017). When the stent is removed during left-sided open-chest surgery, extracorporeal circulation support is required. Moreover, deep-hypothermic circulatory arrest techniques may also be necessary (Lawrence, 2011). During extracorporeal circulation, the recovery of contaminated blood from the surgical field can potentially lead to the dissemination of bacteria and infected tissues throughout the body. In severe cases, septic shock symptoms might occur (Jin et al., 2024). In our surgical strategy, a diversion scheme was adopted, with the ascending aorta diverted to the brachiocephalic artery and the abdominal aorta, respectively. In the cases where there were no aneurysms in the ascending aorta, the ascending aorta and artificial blood vessels were anastomosed in an end-to-side manner. The surgery could be performed at room temperature, thereby avoiding damage associated with extracorporeal circulation and simplifying the surgical process. Moreover, artificial blood vessels could be placed in non-infected areas, effectively preventing the recurrence of infection. It should be noted that one patient with an aortic root aneurysm underwent a Bentall operation, and extracorporeal circulation was established for this patient. Among all patients who recovered, prolonged antibiotic treatment was effectively avoided, significantly improving their quality of life.

In the *in situ* thoracic aortic replacement surgery, it is challenging to remove the infected stent without causing further infection (Uno et al., 2017; Aparicio-Lopez et al., 2023). AEF serves as a nidus for continuous bacterial proliferation and persistent infection. The surgical method used in this study involved removing subsequent infected stents by diverting the brachiocephalic vessels and placing an extra-anatomical bypass graft in a sterile field, which effectively reduced the risk of further infection. Notably, during the treatment of distal anastomosis, it is necessary to cut approximately 1 cm of the intima to ensure communication between the true and false lumens when the descending aorta is accompanied by dissection and both the true and false lumens are required to supply blood to important organs. If the distal end below the renal artery is a stent or an aneurysm, vessel replacement should be carried out first. During the treatment of AEF, most esophageal fistulas were less than 1 cm in size. Thus, local repair using intercostal muscle filling or omentum coverage was commonly used. Most fissures can heal with scarring within a month if adequate nutritional support is provided. However, for fissures larger than 3 cm, the probability of uncontrolled mediastinal infection after surgery remains high. Further research is required to determine whether esophagectomy and secondary reconstruction should be utilized.

For some patients, esophageal stents were utilized to isolate the repaired fistula from the corrosive effects of digestive fluids. However, the stent did not accelerate the healing process. In this study, intravenous sensitive antibiotics were extensively administered for 6–8 weeks, followed by a 2-week course of oral administration. After rechecking various infection indicators, antibiotics could be completely discontinued. During the follow-up period, no recurrence of infection was observed. For patients with severe hematemesis before surgery, a covered stent could be implanted first in emergencies. Nevertheless, such patients typically have larger esophageal fissures and more severe infections, and their prognosis is relatively poor even with subsequent surgical treatment. In this study, fatal mediastinal infections developed among two patients who experienced severe hematemesis and were re-bridged with a covered stent. Thus, the interval between aortic stent placement and surgery should be further evaluated for patients who experience severe hematemesis.

Postoperatively, all patients completed a standardized course of antibiotic treatment based on culture results, and no recurrent infections related to inadequate antibiotic therapy were observed during the follow-up period. Notably, no patients required long-term suppressive antibiotic therapy. This favorable outcome is attributed to the core advantage of our surgical approach: no grafts were implanted in the infected area, which eliminated the persistent nidus of infection that would otherwise necessitate long-term suppressive therapy. The individualized antibiotic protocol, tailored to prior treatment history and culture results, is a critical supporting measure for the success of our surgical strategy. By integrating empirical selection based on prior antibiotic response and culture-directed adjustments, we ensured targeted control of infection, which laid a solid foundation for surgical treatment. Furthermore, the avoidance of long-term suppressive antibiotic therapy not only reduces the risk of antibiotic resistance and adverse drug reactions but also improves patients’ long-term quality of life. This combination of surgical and antibiotic strategies provides a valuable clinical reference for the management of infected AEF.

This study has several limitations that should be acknowledged. First, as a single-center retrospective study with a relatively small sample size, the generalizability of our conclusions may be limited, primarily due to the extremely low incidence of post-TEVAR AEF. Second, the heterogeneity underlying aortic diseases among the study cohort may introduce potential confounding factors and therapeutic responses of post-TEVAR AEF. Third, due to the multi-centric referral nature of the study cohort, comprehensive baseline data regarding the initial TEVAR procedure and long-term follow-up of the initial aortic pathology were not fully verifiable, which may limit the depth of analysis on factors associated with post-TEVAR AEF. Fourth, the absence of a control group treated with classical surgical methods, due to the rarity of the disease, limits the ability to perform a direct comparative efficacy analysis between our strategy and conventional approaches. Future multi-centric, prospective studies with standardized data collection, larger sample sizes, and stratified analysis based on the underlying aortic disease types are required to validate the long-term efficacy of our surgical strategy. Additionally, such studies could help explore the association between the characteristics of initial TEVAR and the incidence of post-TEVAR AEF and provide direct comparative evidence with conventional surgical methods.

## 5 Conclusion

In summary, the approach of aortic arch debranching combined with extra-anatomic aortic bypass can significantly reduce the high rate of infection recurrence and improve prognosis. However, this surgical strategy modifies the normal blood vessel pathway and may lead to infections or other complications related to abdominal artificial blood vessels in the long term. Thus, the accumulation of additional cases and longer-term follow-up are required to validate these findings.

## Data Availability

The original contributions presented in the study are included in the article/Supplementary Material; further inquiries can be directed to the corresponding author.
